# Evaluation of Polysaccharide-Based Latex Agglutination Assays for the Rapid Detection of Antibodies to *Burkholderia pseudomallei*

**DOI:** 10.4269/ajtmh.15-0114

**Published:** 2015-09-02

**Authors:** Vichaya Suttisunhakul, Narisara Chantratita, Chanthiwa Wikraiphat, Vanaporn Wuthiekanun, Zakiya Douglas, Nicholas P. J. Day, Direk Limmathurotsakul, Paul J. Brett, Mary N. Burtnick

**Affiliations:** Department of Microbiology and Immunology, Faculty of Tropical Medicine, Mahidol University, Bangkok, Thailand; Mahidol-Oxford Tropical Medicine Research Unit, Faculty of Tropical Medicine, Mahidol University, Bangkok, Thailand; Department of Microbiology and Immunology, University of South Alabama, Mobile, Alabama; Center for Tropical Medicine and Global Health, Nuffield Department of Medicine, University of Oxford, Oxford, United Kingdom; Department of Tropical Hygiene, Faculty of Tropical Medicine, Mahidol University, Bangkok, Thailand

## Abstract

Melioidosis is a severe disease caused by the Gram-negative bacterium *Burkholderia pseudomallei*. Diagnosis of melioidosis currently relies on the isolation of *B. pseudomallei* from clinical samples, which can take several days. An indirect hemagglutination assay (IHA) is widely used for serodiagnosis, but it has a short shelf life, is poorly standardized, and requires a viable bacteria culture performed in a biosafety level 3 (BSL-3) laboratory. To improve the diagnostic methods, we have developed two rapid latex agglutination tests based on purified *B. pseudomallei* O-polysaccharide (OPS) and capsular polysaccharide (CPS) antigens. The immunodiagnostic potential of these tests was evaluated using serum from culture-confirmed melioidosis patients (*N* = 143) and healthy donors from either endemic (*N* = 199) or non-endemic areas (*N* = 90). The sensitivity of the OPS-based latex agglutination assay (OPS-latex; 84.4%) was significantly higher than both the CPS-latex (69.5%) (*P* < 0.001) and IHA (69.5%) (*P* = 0.001). When evaluated with Thai donor serum, the OPS-latex had comparable specificity (56.9%) to the CPS-latex (63.8%) (*P* = 0.053), but was significantly lower than the IHA (67.6%) (*P* = 0.002). In contrast, all tests with U.S. donor serum were highly specific (≥ 97.8%). These results suggest that polysaccharide-based latex agglutination assays may be useful for serodiagnosis of melioidosis in non-endemic areas.

## Introduction

Melioidosis is a potentially fatal disease caused by the environmental gram-negative bacterium *Burkholderia pseudomallei*. The disease is endemic in tropical countries and continues to be a significant public health concern with increasing numbers of cases reported over the past century in many countries across the world.^1^–^3^ Management of melioidosis patients can be challenging because of the requirement of specific antibiotic treatment with ceftazidime or a carbapenem. In northeast Thailand, the mortality rate of the patients is 40%, and melioidosis is the third most common cause of death from infectious diseases after acquired-immunodeficiency syndrome (AIDS) and tuberculosis.^3^ *Burkholderia pseudomallei* typically infects people following exposure to contaminated soil and water by inoculation, inhalation, or ingestion.^4^ The clinical spectrum of melioidosis is diverse consisting of acute fulminant septicemia, subacute illness, chronic infection, and subclinical disease. Melioidosis is often associated with a prolonged fever and bacteremia, and it can involve multiple organ infections.^5^ In acute forms, death can occur within 24–48 hours of the onset of symptoms.^6^

The standard diagnostic method for melioidosis is bacterial culture followed by biochemical identification. The culture method is specific but has some limitations including that it has low sensitivity and takes several days before a result is available.^7^ The known standard serology test for melioidosis is an indirect hemagglutination assay (IHA) that has been reported to be unreliable in many studies.^8^–^10^ Because of its simplicity to perform, IHA is still widely used for serodiagnosis. Because the IHA is prepared using sheep red blood cells sensitized with crude *B. pseudomallei* antigen, the assay tends to have low specificity and has a limited shelf life. Since the IHA is prepared from viable bacterial cultures, the preparation of the assays in countries where *B. pseudomallei* is categorized as a select agent is neither desirable nor practical. In addition, the IHA is poorly standardized because different strains have been used for antigen preparations in different laboratories.

To address these issues we have developed two latex agglutination assays based on surface-exposed carbohydrate antigens expressed by *B. pseudomallei* for the rapid serodiagnosis of melioidosis. The O-polysaccharide (OPS) component of lipopolysaccharide (LPS) and the 6-deoxy-heptan capsular polysaccharide (CPS) were selected as potential candidate antigens for serodiagnostic tests because they are highly conserved across *B. pseudomallei* strains, but are structurally different from other bacterial pathogens.^11^,^12^ In addition, these are well-characterized polysaccharide antigens, and previous studies have demonstrated that both OPS-specific and CPS-specific antibodies can be detected in melioidosis patient serum.^13^–^15^ In this study, we compared the performance of an OPS-based latex agglutination assay (OPS-latex) and an CPS-based latex agglutination assay (CPS-latex) with the standard IHA for the detection of antibodies to *B. pseudomallei* in serum of melioidosis patients in northeast Thailand and of healthy donors from endemic and non-endemic areas.

## Materials and Methods

### Serum samples.

Three sets of human serum samples were used. The first set consisted of 143 sera from culture-confirmed *B. pseudomallei*-infected patients on admission. The patients were admitted to Sappasithiprasong hospital, Ubon Ratchathani, northeast Thailand. This serum was leftover from a previous study.^16^ The second set was 199 serum samples obtained from healthy donors from the same area in northeast Thailand. The last set was 90 serum samples obtained from healthy U.S. donors (Innovative Research, Novi, MI). All serum samples were stored at −80°C. Freeze and thaw was kept to a minimum. All serum samples were used anonymously for diagnostic development. The study was approved by Ethics Committee of Faculty of Tropical Medicine, Mahidol University.

### Purification of *B. pseudomallei* OPS and CPS.

Broth in 2-L baffled Erlenmeyer flasks was inoculated with the select agent excluded strains *B. pseudomallei* RR2808 (CPS mutant) or RR2683 (OPS mutant) and incubated overnight at 37°C with vigorous shaking. Both of these mutants are derivatives of *B. pseudomallei* Bp82, which expresses type A OPS.^12^,^17^ Cell pellets were obtained by centrifugation and extracted using a modified hot aqueous phenol procedure.^18^ Purified OPS and CPS antigens were then obtained as previously described.^19^,^20^

### Activation of latex beads with *B. pseudomallei* OPS and CPS.

Purified OPS and CPS antigens were solubilized at 5 mg/mL in phosphate buffered saline (PBS; pH 7.2) and added to small amber vials. To each milliliter of the OPS and CPS solutions, 6 mg (30 mM) of sodium *m*-periodate was added. Once the crystals had dissolved, the reaction mixtures were incubated for 40 minutes at room temperature with stirring. To remove any excess oxidizing agent, the reaction mixtures were applied to 10-mL Zeba Desalt Spin Columns (Pierce, Rockford, IL) equilibrated with PBS and the eluates were collected. To facilitate conjugation of the OPS and CPS to amine-derivatized latex beads (1 μm; Life Technologies, Carlsbad, CA), 1.6 mL of each polysaccharide solution (5 mg/mL) was mixed with 8 mL of the latex beads (12.5 mg/mL in PBS) in 15-mL conical tubes. After mixing, 10 μL of 1 M sodium cyanoborohydride stock (in 10 mM NaOH) was added to each milliliter of the conjugation mixtures and the reaction mixtures were incubated at room temperature for 24 hours with gentle mixing on a rotating platform. The latex beads were washed with PBS to remove any unreacted polysaccharide, then incubated overnight with 10 mL of Starting Block T20 (tris-buffered saline [TBS]; Pierce) buffer. The polysaccharide-modified beads were then washed twice with PBS, resuspended in 12 mL of PBS and stored at 4°C until required for use.

### Indirect hemagglutination.

IHA antigens prepared from two clinical *B. pseudomallei* isolates, strains 199a and 207a, were pooled and used to sensitize sheep red blood cells as per established protocols.^21^ Before testing, the serum (50 μL) was inactivated at 56°C for 30 minutes, followed by pre-adsorption with 10% non-sensitized sheep red blood cells in PBS (pH 7.2). Of 1% sensitized and non-sensitized cell controls, 25 μL were incubated with 50 μL of 2-fold serial dilutions of each serum sample in 96-well plates as previously described.^22^ The results were read at the highest antibody titer that showed agglutination after incubating at room temperature for 2 hours. Positive IHA results were determined at a cutoff dilution ≥ 1:160.^21^,^22^

### Latex agglutination tests.

The OPS- and CPS-latex agglutination tests were initially evaluated for sensitivity and specificity using pooled serum from five melioidosis patients or five healthy Thai donors. To perform the assays, 10 μL of OPS-latex beads, CPS-latex beads, or uncoated latex beads were separately mixed with an equal volume of each serum sample on a glass slide and rotated at room temperature for 5 minutes. When conducting the assays, serum was used at a 1:10 dilution (in PBS) for the OPS-latex test and undiluted for the CPS-latex test and uncoated latex beads. The agglutination results were observed by naked eye against a black card and recorded as either positive (agglutination) or negative (no agglutination).

### Statistical analysis.

Statistical analyses were performed using Stata version 12 (StataCorp LP, College Station, TX). The McNemar test was used to compare the sensitivity and specificity between the different tests. The agreement between different tests was determined by Kappa analysis. The nonparametric test for trend of latex agglutination results was performed across the ordered groups of IHA titers. Differences were considered statistically significant if *P* value was ≤ 0.05.

## Results

### Evaluation of polysaccharide-based latex agglutination tests and IHA titers.

**Figure 1.**
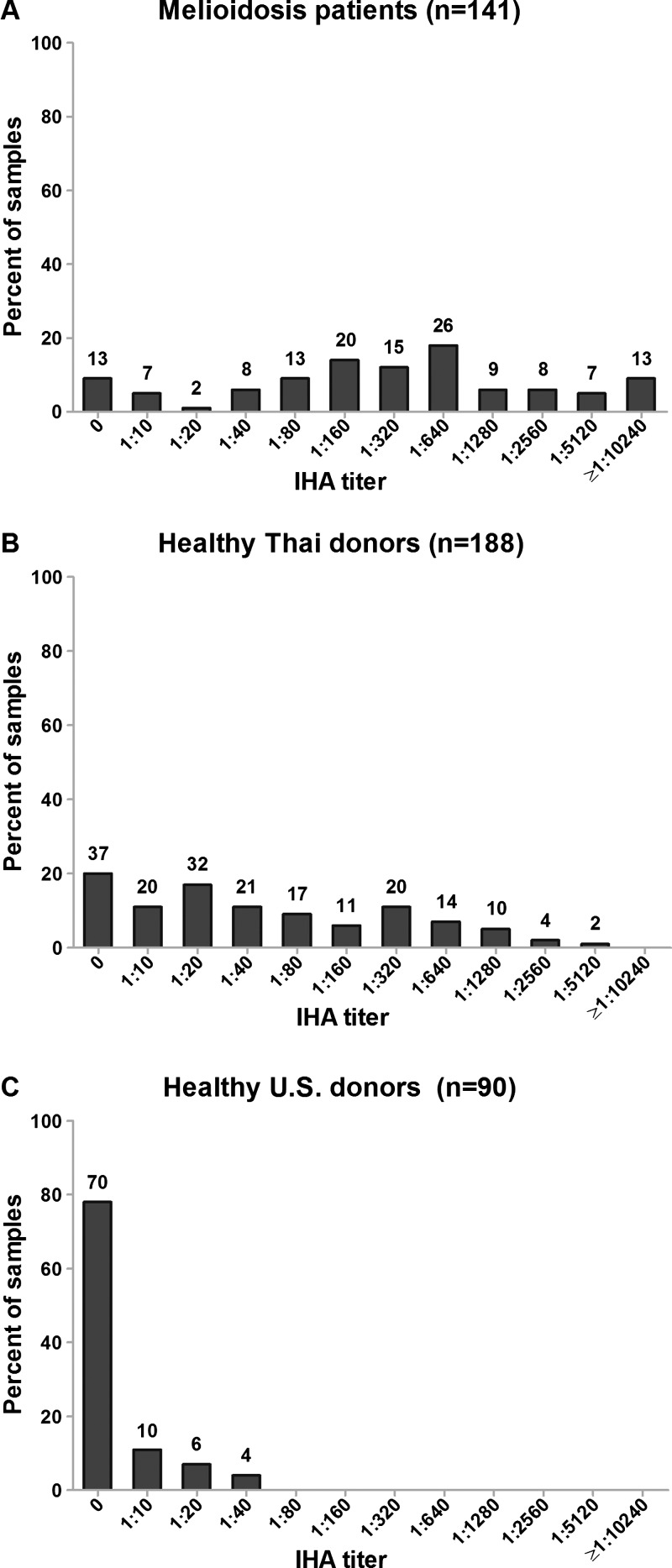
Indirect hemagglutination assay (IHA) titers of melioidosis patients (**A**), healthy Thai donors (**B**), and healthy U.S. donors (**C**).

A diagnostic evaluation of the OPS- and CPS-latex agglutination assays was conducted, and the results were compared with the corresponding IHA titers of the samples tested. Two serum samples from the melioidosis group (*N* = 143) and 11 of the healthy Thai donors (*N* = 199) demonstrated agglutination with the uncoated latex beads (false positives) and were excluded from our analysis. None of the serum from the 90 healthy U.S. donors demonstrated agglutination with the uncoated latex beads. Thus, the serum samples analyzed included 141 patients with culture-confirmed melioidosis, 188 healthy donors from Thailand, and 90 healthy donors from the United States. The melioidosis patients and healthy Thai donors exhibited a wide range of IHA serum titers (Figure 1

). The range for the melioidosis patients was 0 to ≥ 1:10,240 (median = 1:320, interquartile range [IQR] = 1:80–1:1,280) and for the healthy Thai donors was 0 to 1:5,120 (median = 1:40, IQR = 1:10–1:320). In contrast, the IHA titers of the healthy U.S. donors were < 1:80 in all cases, and ranged between 0 and 1:40 (median = 0, IQR = 0).

As shown in Table 1, the diagnostic sensitivity of CPS-latex was comparable to the IHA (69.5%; 98 of 141 patients for both, *P* = 1.000). The sensitivity of the OPS-latex (84.4%; 119 of 141 patients) was significantly higher than the IHA (69.5%; 98 of 141 patients) (*P* = 0.001). When using serum from healthy Thai donors for analysis, the specificity of the CPS-latex (63.8%; 120 of 188 healthy donors) was comparable to the IHA (67.6%; 127 of 188 healthy donors) (*P* = 0.296). In contrast, the specificity of OPS-latex was significantly lower (56.9%; 107 of 188 healthy donors) than the IHA (*P* = 0.002). When evaluated with serum from healthy U.S. donors, all three tests were highly specific. The specificities of both CPS-latex and OPS-latex were 97.8% (88 of 90 healthy U.S. donors) and the IHA was 100%. Only two weakly positive reactions with either the CPS-latex (IHA titer 1:20 for both sera) or OPS-latex (IHA titer 1:10 for both sera) were observed for the healthy U.S. donor samples.

### IHA titers are in agreement with the polysaccharide-based latex agglutination tests.

**Figure 2.**
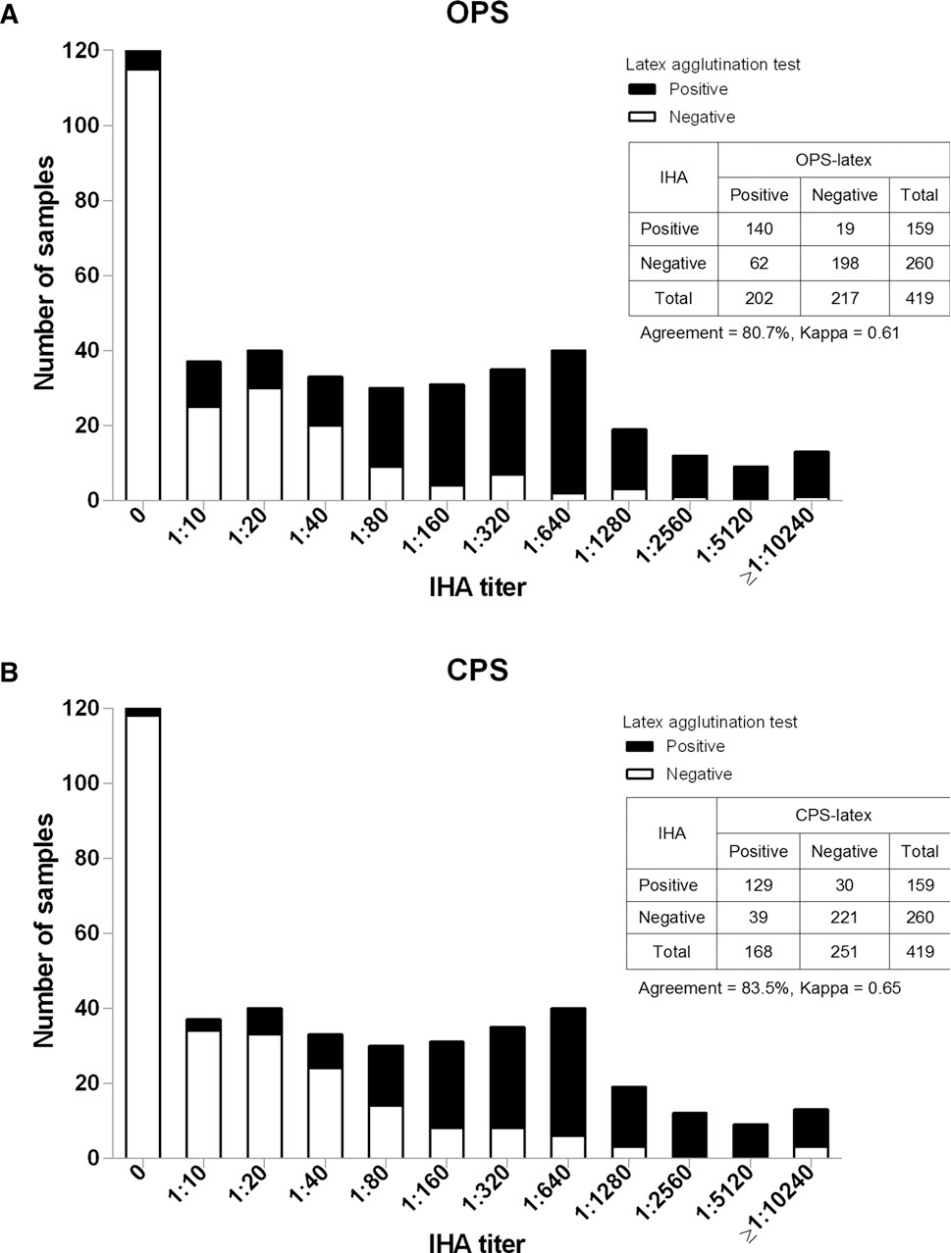
Positive and negative results of O-polysaccharide (OPS)–latex agglutination test (**A**) and capsular polysaccharide (CPS)–latex agglutination test (**B**) of all serum samples compared with indirect hemagglutination assay (IHA) titers.

Kappa analysis of all samples showed that the results of OPS- and CPS-latex were in good agreement with the IHA results (agreement 80.7%, kappa 0.61, *P* < 0.001 for OPS-latex and agreement 83.5%, kappa 0.65, *P* < 0.001 for CPS-latex). There were trends in OPS- and CPS-latex results across the ordered levels of IHA titers (*P* < 0.001 for both) (Figure 2

). Interestingly, we observed a prozone phenomenon that showed false negative latex agglutination resulting from overwhelming antibody IHA titers at ≥ 1:10,240 (1 of 13 patients for OPS-latex and 3 of 13 patients for CPS-latex) (Figure 2). Dilution of these serum samples by 2- to 4-fold resulted in positive agglutination reactions.

## Discussion

Although definitive diagnosis of melioidosis requires bacterial culture and further identification; serological test results may be useful to provide evidence of infection and/or exposure to *B. pseudomallei*. Using serum from culture-confirmed melioidosis patients, healthy Thai donors, and healthy U.S. donors, we have demonstrated that both the OPS- and CPS-latex tests developed in this study provide results that are comparable to the IHA that is currently the most commonly used serological test for melioidosis. Consistent with previous reports, our findings suggest that the high rates of seropositivity in healthy individuals from endemic areas influence the specificity of these assays. The reason for this phenomenon is unclear but it is likely that individuals in these regions have a high incidence of exposure to *B. pseudomallei* or to antigenically related organisms that are ubiquitous in the environment.^10^,^22^

The results obtained from both polysaccharide-based latex agglutination tests were in good agreement with the results obtained by the IHA. Although the antigens in the IHA are undefined, our finding suggests that these polysaccharides may be two of the major antigens in this assay. Although the CPS-latex performed equivalently to the IHA, the OPS-latex had higher sensitivity, but lower specificity. At present, it is not clear why the OPS-latex showed higher sensitivity than the CPS-latex and IHA. It is possible that this may be related to the OPS-specific antibody titers in the serum samples since the LPS of *B. pseudomallei* is known to induce strong immune responses in humans.^23^ It would be useful to compare and contrast the new tests and IHA regarding their abilities to detect antibody responses during the course of active infections. Additional studies will be necessary to address this issue.

The low specificity of all three tests with the healthy Thai serum samples suggests that agglutination methods are not ideal for serodiagnosing melioidosis in endemic areas. However, our results indicate that the OPS- and CPS-latex may be useful for assessing seropositivity and/or exposure events in individuals from non-endemic areas. Benefits of the polysaccharide-based latex agglutination tests include that they have a longer shelf life than the IHA, are inexpensive, standardized, and simple to perform. Some limitations of latex tests include the following: 1) non-specific agglutination of some of the serum samples with uncoated latex beads suggests that inclusion of uncoated beads may be required as a control, 2) false negative responses associated with prozone effects, and 3) the use of only type A OPS, which may result in false negatives in patients infected with *B. pseudomallei* strains that have different LPS serotypes (e.g., type B or B2 or rough type).^17^ Collectively, our findings suggest that CPS and OPS antigens are potentially valuable diagnostic reagents that may be useful for rapidly detecting *B. pseudomallei* infections. Further development of alternative assay formats should be considered to help eliminate issues associated with false positive results when used in endemic areas.

## Figures and Tables

**Table 1:** Sensitivity and specificity of OPS- and CPS-latex agglutination tests and IHA

Test	Cutoff	% Sensitivity (95% CI)	% Specificity (95% CI)	
		Melioidosis	Thai donors	U.S. donors
		*N* = 141	*N* = 188	*N* = 90
OPS-latex	Agglutination	84.4 (77.3–90.0)	56.9 (49.5–64.1)	97.8 (92.2–99.7)
CPS-latex	Agglutination	69.5 (61.2–77.0)	63.8 (56.5–70.7)	97.8 (92.2–99.7)
IHA	Titer ≥ 1:160	69.5 (61.2–77.0)	67.6 (60.4–74.2)	100 (96.0–100)

CI = confidence interval; CPS = capsular polysaccharide; IHA = indirect hemagglutination assay; OPS = O-polysaccharide.
